# Effects of storage time and temperature on coagulation factor and natural anticoagulant activities in healthy individuals

**DOI:** 10.1038/s41598-025-95389-w

**Published:** 2025-03-28

**Authors:** Ryosuke Nakanishi, Tasuku Sakayori, Daichi Matsui, Mai Kono, Minase Maki, Claire Dunois, Yutaka Komiyama, Hiromi Morisaki

**Affiliations:** 1 Medical & Scientific Affairs, Sysmex Corporation, Kobe, 651-2271 Japan; 2 Global Management, Sysmex Corporation, 1-3-2 Murotani, Nishi-ku, Kobe, Hyogo 651-2241 Japan; 3 Validation and Clinical Studies, HYPHEN BioMed, 95000 Neuville sur Oise, France; 4 Faculty of Health and Medical Sciences, Hokuriku University, Kanazawa, 920-1180 Japan; 5 Corporate Business Planning, Sysmex Corporation, Kobe, 651-0073 Japan

**Keywords:** Pre-analytical variable, Coagulation tests, Stability, Storage conditions, Clinical and laboratory standards Institute, Coagulation system, Assay systems, Biochemical assays, Diagnostics, Laboratory techniques and procedures, Medical and clinical diagnostics

## Abstract

Coagulation test results are influenced by many pre-analytical variables, including clinical sample storage conditions. This study investigated the effects of storage time and temperature on coagulation factor (FII, FV, FVII, FX, FVIII, FIX, FXI, FXII, and FXIII) and natural anticoagulant (antithrombin, protein C, and free protein S) activities. Blood samples were collected from 78 healthy individuals, and 3 samples were used for each coagulation factor and natural anticoagulant. Plasma was tested after centrifugation and storage at room temperature (18 to 25 °C) or refrigerated (2 to 8 °C) for 2, 3, 4, and 5 h, or frozen (− 15 to − 25 °C) for 1, 2, 3, and 4 months. For all storage conditions, the mean percentage change from baseline in evaluated activities was < 15%, except for FV and FVIII. For FV activities after frozen storage at − 15 to − 25 °C and FVIII activities at all storage conditions, the mean percentage change was > 15%. For FV and FVIII activities, the mean percentage change was < 15% after frozen storage at − 75 to − 85 °C for 1, 2, 3, and 4 months. To ensure appropriate clinical testing results, especially for FV and FVIII activity, storage condition and time should be critically controlled.

**Supplementary Information:**

The online version contains supplementary material available at 10.1038/s41598-025-95389-w.

## Introduction

Numerous pre-analytical variables may influence coagulation test results. The most important pre-analytical variables include sample storage time and temperature^1,2^. Erroneous results, influenced by pre-analytical variables, can significantly impact diagnosis and treatment, and consequently patient outcomes^3^.

The Clinical and Laboratory Standards Institute (CLSI) H21-ED6 guideline recommends acceptable storage times for plasma samples at various storage conditions (room temperature, refrigerated, or frozen) (Table 1)^4^. The main text of the CLSI H21-ED6 guideline states that samples for prothrombin time (PT), fibrinogen, and D-dimer tests can be stored at room temperature for up to 24 h after blood collection, and samples for activated partial thromboplastin time (APTT) (not for heparin monitoring) can be stored at room temperature for up to 8 h. For other coagulation factor and natural anticoagulant activities, the CLSI H21-ED6 guideline suggests that additional studies are needed to determine whether extending the acceptable storage time beyond the existing default of 4 h is safe. However, evidence provided in the CLSI H21-ED6 guideline is limited. Appendix D of the CLSI H21-ED6 guideline lists the acceptable storage times for each parameter (coagulation factors and natural anticoagulants) at room temperature and when refrigerated, excluding factor XIII (FXIII), antithrombin (AT), protein C (PC), and free protein S (PS). However, this can be considered preliminary evidence.

**Table 1:** Comparison of acceptable storage times for FII, FV, FVII, FX, FVIII, FIX, FXI, FXII, FXIII, AT, PC, and free PS activities between the CLSI guideline and this study.

Factor	Acceptable storage time (main text of the CLSI H21-ED6 guideline)				Acceptable storage time (this study)			
	Room temperature (15–25 °C)	Refrigerated (2–8 °C)	Frozen (≤ − 20 °C)	Frozen (≤ − 70 °C)	Room temperature (18–25 °C)	Refrigerated (2–8 °C)	Frozen (− 15 to − 25 °C)	Frozen (− 75 to − 85 °C)
FII (%)	4 h	4 h	3 months	18 months	5 h	5 h	4 months	Not tested
FV (%)	4 h	4 h	3 months	18 months	5 h	5 h	unacceptable	4 months
FVII (%)	4 h	4 h	3 months	18 months	5 h	5 h	4 months	Not tested
FX (%)	4 h	4 h	3 months	18 months	5 h	5 h	4 months	Not tested
FVIII (%)	4 h	4 h	3 months	18 months	3 h	4 h	1 month	4 months
FIX (%)	4 h	4 h	3 months	18 months	5 h	5 h	4 months	Not tested
FXI (%)	4 h	4 h	3 months	18 months	5 h	5 h	4 months	Not tested
FXII (%)	4 h	4 h	3 months	18 months	5 h	5 h	4 months	Not tested
FXIII (%)	4 h	4 h	3 months	18 months	5 h	5 h	4 months	Not tested
AT (%)	4 h	4 h	3 months	18 months	5 h	5 h	4 months	Not tested
PC (%)	4 h	4 h	3 months	18 months	5 h	5 h	4 months	Not tested
Free PS (%)	4 h	4 h	3 months	18 months	5 h	5 h	4 months	Not tested

*FII* factor II, *FV* factor V, *FVII* factor VII, *FX* factor X, *FVIII* factor VIII, *FIX* factor IX, *FXI* factor XI, *FXII* factor XII, *FXIII* factor XIII, *AT* antithrombin, *PC* protein C, *Free PS* free protein S.

Few studies have suggested acceptable storage times at room temperature and under refrigeration for coagulation factor activities^5–8^. Previous studies have indicated that the acceptable storage times at room temperature and under refrigeration for FV and FVIII activities are shorter than those for other coagulation factors^5–8^.

When samples are frozen, acceptable storage times for PT, APTT, fibrinogen antigen, and Clauss fibrinogen have been considered in the main text of the CLSI H21-ED6 guideline. The CLSI H21-ED6 guideline recommends that plasma samples, when used for coagulation testing, be stored frozen for up to 3 months at ≤ -20 °C and up to 18 months at ≤ − 70 °C. However, the evidence supporting these recommendations is limited. The acceptable storage times for each parameter (coagulation factors and natural anticoagulants), excluding FXIII and free PS are detailed in Appendix D of the CLSI H21-ED6 guideline. However, the CLSI H21-ED6 guideline states that these acceptable storage times may exceed the general recommendations for frozen storage.

Previous studies have reported that acceptable frozen storage times for FVIII and FIX activities are shorter than those for other coagulation tests^9^. However, the findings of the few studies on frozen storage of samples must be validated.

To ensure accurate measurement data using a combination of reagents and equipment, sample stability must be considered. This study investigated the effects of storage time and temperature, including room temperature (18 to 25 °C), refrigerated (2 to 8 °C), and frozen (− 15 to − 25 °C) storage, in the clinical laboratory environment on coagulation factor (FII, FV, FVII, FX, FVIII, FIX, FXI, FXII, and FXIII) and natural anticoagulant (AT, PC, and free PS) activities.

## Results

### Trend of coagulation factor and natural anticoagulant stability under different storage conditions

**Fig. 1:**
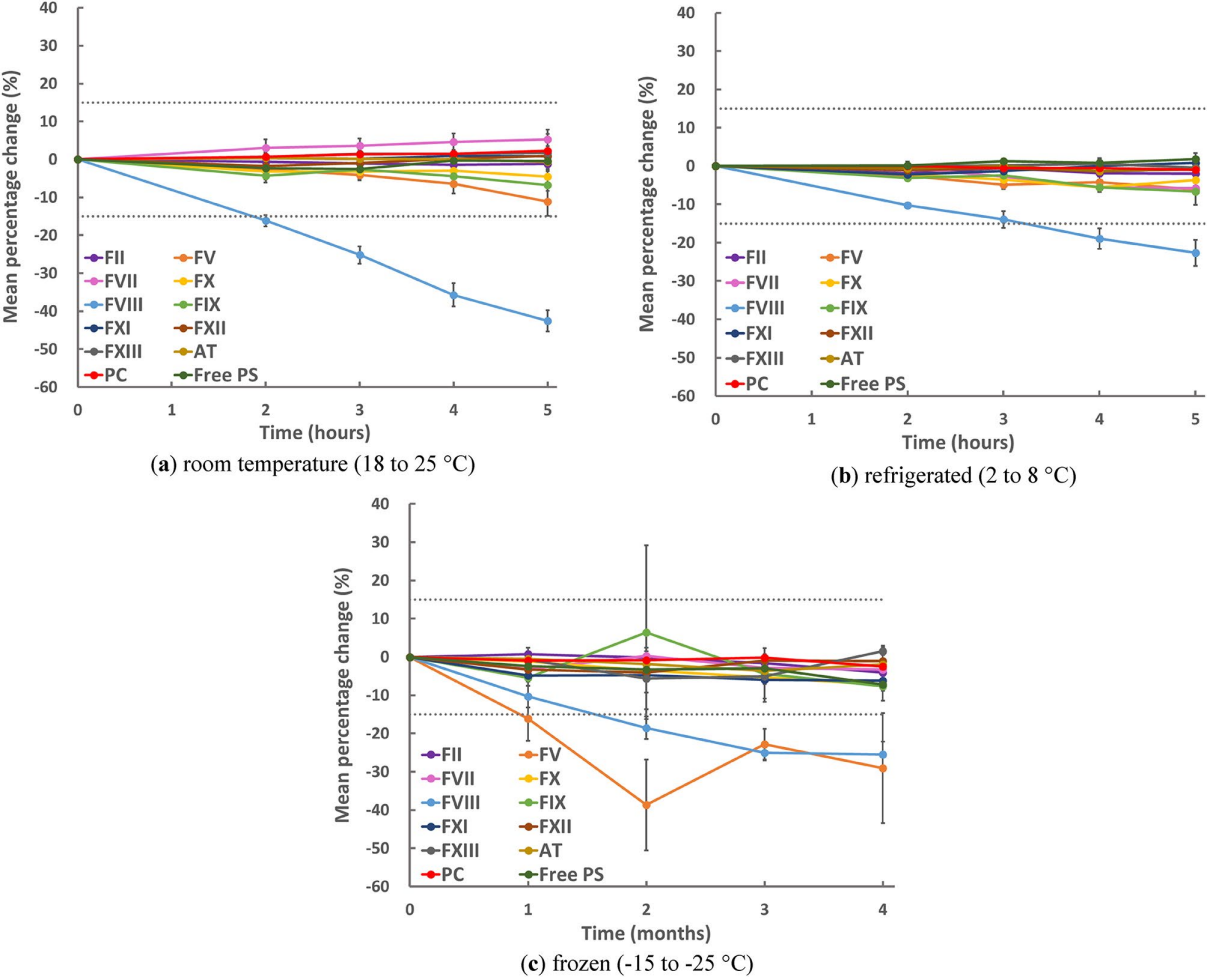
Trend plot for mean percentage changes in coagulation and anticoagulant factor activities in sample after storage. Trend plot for mean percentage changes in FII, FV, FVII, FX, FVIII, FIX, FXI, FXII, FXIII, AT, PC, and free PS activities in samples after storage for 2, 3, 4, and 5 h at room temperature (18 to 25 °C) (**a**); 2, 3, 4, and 5 h under refrigeration (2 to 8 °C) (**b**); and 1, 2, 3, and 4 months at frozen (− 15 to − 25 °C) (**c**), compared with the baseline results. The two dotted lines in the figures represent ± 15%. *FII* factor II, *FV* factor V, *FVII* factor VII, *FX* factor X, *FVIII* factor VIII, *FIX* factor IX, *FXI* factor XI, *FXII* factor XII, *FXIII* factor XIII, *AT* antithrombin, *PC* protein C, *Free PS* free protein S.

Figure 1 presents the trend plot for the mean percentage changes in FII, FV, FVII, FX, FVIII, FIX, FXI, FXII, FXIII, AT, PC, and free PS activities after room temperature (Fig. 1a) or refrigerated (Fig. 1b) storage for 2, 3, 4, and 5 h, or after frozen (− 15 to − 25 °C) (Fig. 1c) storage for 1, 2, 3, and 4 months, compared with their respective baseline values (0 h and 0 months). Supplementary Table 1 shows the values and statistical differences in these activities after storage at each temperature.

### Room temperature and refrigerated storage

After room temperature (Fig. 1a) or refrigerated (Fig. 1b) storage, the mean percentage changes in all factor and natural anticoagulant activities, except FVIII, were less than 15% within the time frame of this study. FV activity after storage for 5 h at room temperature decreased from 107 to 95%, and the mean percentage change was − 11%. The stability of FVIII activity after room temperature and refrigerated storage was lower than that of the activities of other factors. The mean percentage changes in FVIII activity after room-temperature storage for more than 2 h or after refrigerated storage for more than 4 h exceeded 15%, with a decrease in FVIII activity from 110 to 92% when stored for 2 h at room temperature and from 110 to 89% when refrigerated for 4 h. The mean percentage changes were − 16% and − 19%, respectively. FVIII activity after room temperature or refrigerated storage for 5 h decreased from 110 to 63% and 85%, respectively. The mean percentage changes were − 43% and − 23%, respectively. The time from blood collection to baseline measurement, including centrifugation time, was approximately 2.5 h.

Since the mean percentage change in FVIII activity after room temperature or refrigerated storage exceeded 15%, additional measurements were conducted every 0.5 h between 1 and 4.5 h under the 2 conditions of storage to investigate the detailed stability of FVIII activity. In these FVIII measurements, the baseline measurement was completed approximately 1.5 h after blood collection, including centrifugation time, due to the small total number of samples for additional measurements.

**Fig. 2:**
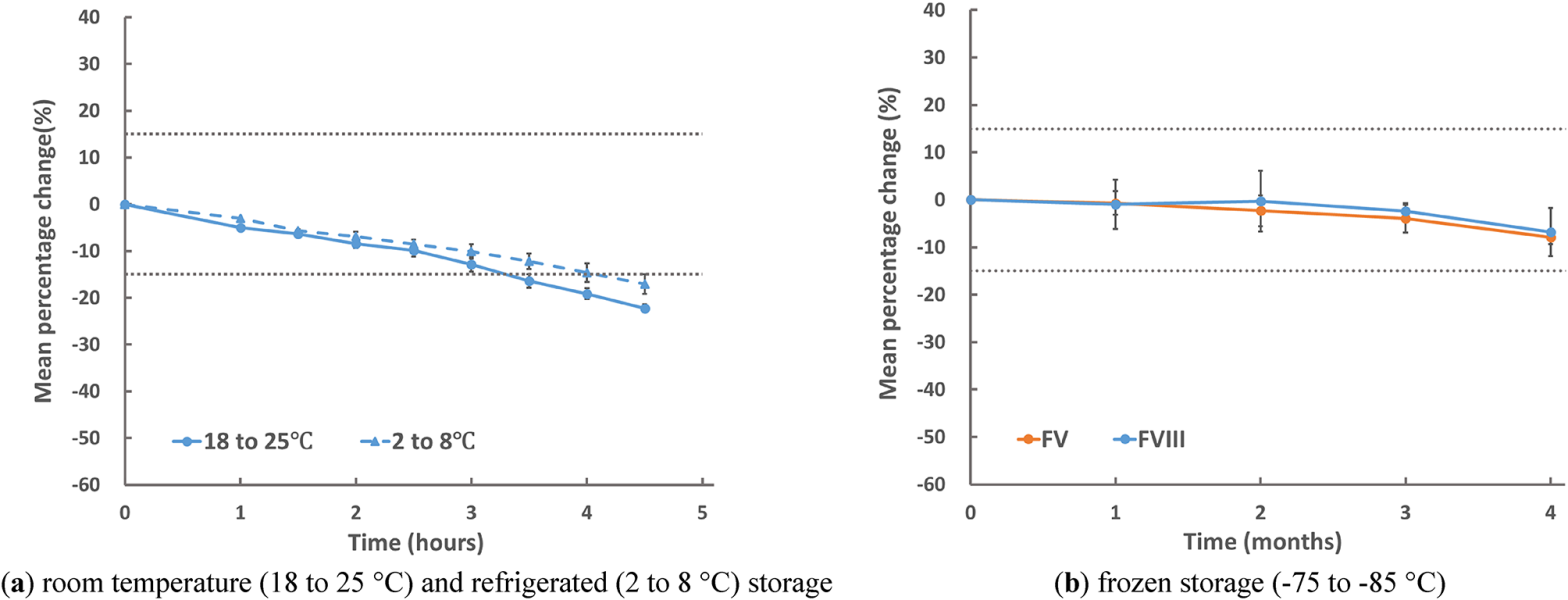
Trend plot for mean percentage changes in FV and FVIII activities in additional samples after storage. (**a**) Trend plot for mean percentage changes in FVIII activities in additional samples after storage for 1, 1.5, 2, 2.5, 3, 3.5, 4, and 4.5 h at room temperature (18 to 25 °C) and under refrigeration (2 to 8 °C), compared with the baseline results. (**b**) Trend plot for mean percentage changes in FV and FVIII activities in samples after frozen (− 75 to − 85 °C) storage for 1, 2, 3, and 4 months, compared with the baseline results. The two dotted lines in the figures represent ± 15%. *FV* factor V, *FVIII* factor VIII.

Figure 2a shows the trend plot for the mean percentage changes in FVIII activity after room-temperature or refrigerated storage for 1, 1.5, 2, 2.5, 3, 3.5, 4, and 4.5 h. The mean percentage change in FVIII activity after room-temperature storage for 3 h or after refrigerated storage for 4 h was less than 15%. FVIII activity after room-temperature storage for 3.5 h decreased from 137 to 115%, and the mean percentage change was − 16%. FVIII activity after refrigerated storage for 4.5 h decreased from 137 to 114%, and the mean percentage change was − 17%. The values and statistical differences in FVIII activity after room-temperature or refrigerated storage for 1, 1.5, 2, 2.5, 3, 3.5, 4, and 4.5 h, are shown in Supplementary Table 2.

### Frozen storage

After frozen storage (− 15 to − 25 °C) (Fig. 1c), the mean percentage changes for all coagulation factor activities and natural anticoagulant activities, except FV and FVIII activities, were less than 15% within the time frame of this study. The stability of FV and FVIII activities after frozen storage (− 15 to − 25 °C) was lower than that of other factor activities. The mean percentage change in FV activity after frozen (− 15 to − 25 °C) storage for more than 1 month and FVIII activity after frozen (− 15 to − 25 °C) storage for more than 2 months exceeded 15%. FV activity after 1 month of frozen storage (− 15 to − 25 °C) decreased from 79 to 66%, with a mean percentage change of − 16%. FVIII activity after 2 months of frozen storage (− 15 to − 25 °C) decreased from 151 to 123%, with a mean percentage change of − 19%.

Therefore, to investigate the detailed stability of FV and FVIII activity after frozen storage, additional measurements were conducted on the samples after frozen storage (− 75 to − 85 °C). Figure 2b shows the trend plot for the mean percentage changes in FV and FVIII activities after frozen storage at ≤ − 75 °C for 1, 2, 3, and 4 months. The mean percentage changes in FV and FVIII activities measured after frozen storage at ≤ − 75 °C for 1, 2, 3, and 4 months were less than 15% for all times. FV and FVIII activities after frozen storage (− 75 to − 85 °C) for 4 months decreased from 113 to 104% and from 137 to 127%, respectively. The samples for FV and FVIII can be safely stored in this time frame when plasma is stored frozen at ≤ − 75 °C. All results and statistical differences in FV and FVIII activities after frozen storage (− 75 to − 85 °C) for 1, 2, 3, and 4 months are shown in Supplementary Table 2.

## Discussion

Table 1 presents a summary of the acceptable storage times according to the main text of the CLSI H21-ED6 guideline and our findings. First, we focused on room-temperature and refrigerated storage. As shown in Table 1, the acceptable storage time at room temperature for FVIII activity, an important laboratory test parameter for the diagnosis of hemophilia and related disorders, was shorter than that recommended in the main text of the CLSI H21-ED6 guideline.

The acceptable room-temperature and refrigerated storage times for FVIII activity were shorter than those for other factors, as previously reported in studies using reagents and equipment from other manufacturers^5,7,8^. The storage times for FVIII activity in previous studies were shorter than the acceptable storage time described in the main text of the CLSI H21-ED6 guideline, supporting the results of the present study. However, this study confirmed that the acceptable room-temperature and refrigerated storage times for FV activity are the same as the acceptable storage times for other coagulation factor and natural anticoagulant activities, excluding FVIII activity. However, FV activity after storage at room temperature for 5 h decreased the most among coagulation factor and natural anticoagulant activities, excluding FVIII activity. FV activity at room temperature tended to decrease. FV activity has long been thought to be unstable with respect to storage time and temperature in clinical laboratory tests. A recent study using other manufacturers’ reagents and equipment demonstrated that the acceptable storage time for FV activity at room temperature is shorter than that of other coagulation factors (FII, FVII, FX, FIX, FXI, and FXII)^8^.

The mean percentage change in FVIII activity after storage for 5 h at room temperature in this study was − 43%, indicating that the activity decreased by almost half. Further studies are necessary for samples with originally low activity, such as hemophilia samples. However, our results suggest that pre-analytical factors might limit diagnostic accuracy in patients with hemophilia.

Next, this study found that storing plasma frozen at − 15 to − 25 °C is not suitable for the stability of FV and FVIII activities. The main text of the CLSI H21-ED6 guideline recommends storing plasma samples frozen at ≤ − 20 °C for up to 3 months. However, since the evidence supporting this recommendation is limited, the stability of plasma samples during frozen storage was compared with findings from other studies. Previous studies using other manufacturers’ reagents have suggested that FVIII and FIX activities decreased when plasma is stored frozen at − 20 °C^9^. FIX activity was stable within the timeframe of this study, whereas FV and FVIII activities were more unstable than those of the other coagulation factors and natural anticoagulants. Although further studies, such as those including larger sample sizes, using patient samples, or using other manufacturers’ reagents, are necessary, based on the results of previous studies and this study, caution must be exercised when storing samples for FV, FVIII, and FIX activity testing at freezing temperatures.

For FV and FVIII activities, which were not stable when plasma was frozen at − 15 to − 25 °C, we changed the storage temperature from freezing at − 15 to − 25 °C to freezing at − 75 to v85 °C, and we obtained additional data. From the results shown in Fig. 2b, FV and FVIII activities after frozen storage at − 75 to − 85 °C were stable within the time frame of this study. While the CLSI H21-ED6 guideline does not mention this, the present study recommends storing samples in a deep freezer for measuring FV and FVIII activities when the sample is stored frozen.

In terms of long-term storage, our results differ from those of a previous study, which reported that FV and FVIII activities were stable for 6 and 3 months, respectively, after frozen storage at − 24 ± 2 °C^10^. However, the previous study involved samples collected via plasmapheresis, which differs from the sample collection method used in the present study. The blood collection method used in this study (brachial vein blood collection) was similar to that commonly used in routine clinical laboratory tests. The differences in blood collection methods are assumed to have influenced the results. Although further studies on the stability of FV and FVIII activities are required, long-term storage is not recommended. To obtain appropriate data, measurements must be performed within a short storage period after blood collection.

Moreover, new insights into the effects of storage time and temperature on coagulation factors and natural anticoagulants were obtained for FXIII (room temperature, refrigerated, and frozen), AT (room temperature and refrigerated), PC (room temperature and refrigerated), and free PS (room temperature, refrigerated, and frozen), which have not been previously reported. The coagulation factors and natural anticoagulants remained stable within these timeframes under all storage conditions.

This study found that the acceptable storage time and temperature varied for each coagulation factor and natural anticoagulant. To provide appropriate measurement data in clinical practice, storage times and temperatures should be critically controlled.

This study has some limitations. First, this study used plasma samples from a limited number of healthy individuals (*n* = 3) for each coagulation factor and natural anticoagulant. Furthermore, the stability of coagulation factor and natural anticoagulant activities may be influenced by patients’ clinical states or medications. Second, only one type of assay, either the chromogenic method or clotting method, was used. Lastly, this was a single-center study. Therefore, the conclusions of this study should be substantiated by further multi-center studies involving different study populations and various types of assays.

In summary, this study showed that samples for the measurement of FII, FV, FVII, FX, FIX, FXI, FXII, FXIII, AT, PC, and free PS activities can be stored for up to 5 h at room temperature or under refrigeration, whereas samples for the measurement of FVIII activity can be stored for only 3 and 4 h, respectively, at the same temperatures. Also, our findings indicate that samples for the measurement of FII, FVII, FX, FIX, FXI, FXII, FXIII, AT, PC, and free PS activities could be stored frozen at − 15 to − 25 °C for up to 4 months. FV and FVIII activities required frozen storage at − 75 to − 85 °C for the same duration.

## Methods

### Ethics statement

This study was approved by the Sysmex Corporation Ethics Committee (approval No., 2021-24). All methods were performed in accordance with Ethical Guidelines for Medical and Biological Research Involving Human Subjects. The internal volunteers provided written informed consent for the use of their samples in this study.

### Participating centers

This study was conducted at Sysmex Corporation (Kobe, Japan). Samples were measured using AUTOMATED BLOOD COAGULATION ANALYZER CS-5100 (Sysmex Corp., Kobe, Japan) and Automated Blood Coagulation Analyzer CN-6000 (Sysmex Corp., Kobe, Japan) with Sysmex (Sysmex Corp., Kobe, Japan) and HYPHEN reagents (HYPHEN BioMed, Paris, France).

### Plasma samples

Healthy individuals (*n* = 78) were recruited from internal volunteers at the Sysmex Corporation for coagulation factors (FII, FV, FVII, FX, FVIII, FIX, FXI, FXII, and FXIII) and natural anticoagulants (AT, PC, and free PS). Plasma samples were prepared according to the consensus established by Ieko et al.^11^. From each volunteer, 9 mL (4.5 mL × 2) blood samples were collected in Venoject II^®^ tubes (Terumo Corp, Tokyo, Japan) containing one-tenth volume of 0.105 M buffered sodium citrate (approximately 3.2%). Whole blood was centrifuged at 1500 × *g* for 15 min at room temperature. The plasma obtained was collected using plastic pipettes and transferred to polystyrene tubes, which were then capped. For each coagulation factor and natural anticoagulant, one group of samples (*n* = 3) was divided between room temperature (18 to 25 °C) and refrigerated storage (2 to 8 °C), while the other group (*n* = 3) was frozen at − 15 to − 25 °C and stored at the same temperature. A portion of each sample was analyzed, and the results served as the baseline (0 h or 0 months). The other samples were tested under the following conditions from baseline measurement: after storage for 2, 3, 4, and 5 h at room temperature and under refrigeration and for 1, 2, 3, and 4 months at frozen (− 15 to − 25 °C). A total of 72 samples were analyzed in these tests. The refrigerators and freezers used to store the samples were monitored. The samples stored frozen at − 15 to − 25 °C were thawed in a water bath for 15 min at 37 °C before use.

If the mean percentage change in the coagulation factor activity tested after storage at room temperature or under refrigeration, compared with the baseline results, did not indicate acceptable storage, additional samples (*n* = 3) were collected for the coagulation factor activity test and measured after every 30 min for 1–4.5 h at the same storage temperature. When the mean percentage change in coagulation factor activities after frozen storage (− 15 to − 25 °C), compared with the baseline results, was deemed unsatisfactory, additional samples (*n* = 3) were collected for the coagulation factor activities test, frozen at − 75 to − 85 °C, and measured after storage for 1, 2, 3, and 4 months at the same temperature. If the mean percentage change in additionally measured results, compared with baseline results, did not indicate acceptable storage, the measurement was considered complete. The freezers were monitored while storing the samples. The samples stored frozen at − 75 to − 85 °C were thawed in a water bath for 15 min at 37 °C before use.

### Assays

The plasma samples were measured using the chromogenic method for FVIII, FXIII, AT, PC, and free PS and the clotting method for FII, FV, FVII, FX, FIX, FXI, and FXII. Measurements were conducted using CS-5100 for AT and CN-6000 for FII, FV, FVII, FX, FVIII, FIX, FXI, FXII, FXIII, PC, and free PS. HYPHEN BioMed reagents included Prothrombin Deficient Plasma, Factor V Deficient Plasma, Factor VII Deficient Plasma, Factor X Deficient Plasma, PT-Phen™ LRT, Revohem™ FVIII Chromogenic, Factor IX Deficient Plasma, Factor XI Deficient Plasma, Factor XII Deficient Plasma, BIOPHEN™ Factor XIII, Revohem™ AT, Revohem™ Protein C, and LIAPHEN™ Free Protein S. Sysmex reagents included Revohem™ APTT SLA and Revohem™ 0.025 M Calcium Chloride Solution. The results are expressed as percentages.

### Statistical analyses

The measured coagulation factor and natural anticoagulant activities are reported as mean ± standard deviation (SD). The statistical significance of differences in values for repeated-measure multiple groups was evaluated using repeated-measures analysis of variance. To assess the stability of these activities, the percentage changes from the baseline values were calculated ([result at storage time X − result at baseline]/result at baseline) and averaged at each time point. A mean percentage change of ≤ 15% compared to the baseline values was considered indicative of acceptable storage^12^. Statistical significance was set at *P* < 0.05. Statistical analyses were performed using JMP 18 (SAS Institute Inc., Cary, NC, USA).

## Electronic supplementary material

Below is the link to the electronic supplementary material.

Supplementary Material 1

## Data Availability

The datasets analyzed during this study are available from the corresponding author on reasonable request.
